# Maternal exposure to oil spill and children’s mental health: The mediating role of resource loss

**DOI:** 10.1371/journal.pone.0335995

**Published:** 2026-06-17

**Authors:** Ariane Lisann Rung, Katie M. Sternberger, Evrim Oral, Edward S. Peters

**Affiliations:** 1 Epidemiology Department, College of Public Health, University of Nebraska Medical Center, Omaha, Nebraska, United States of America; 2 School of Medicine, Louisiana State University Health Sciences Center-New Orleans, New Orleans, Louisiana, United States of America; 3 Biostatistics and Data Science Program, School of Public Health, Louisiana State University Health Sciences Center-New Orleans, New Orleans, Louisiana, United States of America; Universiti Pertahanan Nasional Malaysia, MALAYSIA

## Abstract

**Objectives:**

To assess the effect of maternal oil spill exposure on children’s mental health and examine whether maternal resource loss mediated this relationship.

**Methods:**

445 mother-child pairs from the Women and Their Children’s Health Study in Louisiana (2012–2016) were examined. Maternal oil spill exposure from the 2010 Deepwater Horizon Oil Spill was measured at Wave 1 (2012–2014), while children’s mental health and maternal resource loss were assessed at Wave 2 (2014–2016). Structural equation modeling estimated the direct and indirect effects of maternal oil spill exposure on children’s mental health through maternal resource loss.

**Results:**

Mothers (mean age 42 years (SD 7.7) and children (mean age 13 years (SD 2.2)) were interviewed. Maternal oil spill exposure was not directly related to children’s mental health (est = −0.08, p = 0.436). However, exposure was associated with increased maternal resource loss (est = 0.45, p < .0001), which was associated with worse children’s mental health (est = 0.27, p < .0001). Maternal oil spill exposure was compatible with an indirect negative effect on children’s mental health (i.e., worse mental health scores) mediated by resource loss (est = 0.12, p < 0.005).

**Conclusion:**

Findings suggest maternal oil spill exposure indirectly affects children’s mental health through resource loss. Interventions mitigating resource loss are vital for supporting children’s mental well-being after disasters.

## Introduction

Technological disasters, including oil spills, impact not only the environment and the individuals directly involved, but the neighboring communities as well. The British Petroleum Deepwater Horizon oil platform exploded on April 20, 2010, killing 11 workers and causing the largest marine oil spill in history. The spill, which lasted for 87 days, contaminated over 1,300 miles of Gulf of Mexico shoreline and underwater habitats as deep as 3,600 feet [1].

The mental health impacts of these spills on people living in the surrounding communities are increasingly well-documented and include stress, depression, anxiety, and PTSD [2–6], with the greatest effects resulting from the disruption of lives, work, family, and social engagement, as well as economic loss and commercial ties to renewable resources [7,8]. Studies in Louisiana after the Deepwater Horizon Oil Spill (DHOS) found that among women, more exposure to the oil spill was associated higher levels of depression, mental distress, and domestic conflict [9], with depression and mental distress persisting even 4–6 years after the spill [10].

Although many studies have explored the mental health effects of oil spills and other disasters on adults, considerably fewer have focused on children’s mental health. A study following the 2007 Hebei Spirit Oil Spill indicated that children residing closer to affected areas showed significantly higher levels of depression compared to those residing farther away [11]. In the aftermath of the Deepwater Horizon Oil Spill in 2010, nearly 20% of Gulf Coast parents observed signs of both physical and mental health issues in their children [12], and children who were directly exposed through physical contact were significantly more likely to experience physical and mental health issues compared to their unexposed counterparts [13]. Subsequent research on youth found that stressors related to the DHOS were common and associated with PTSD symptoms [14].

Children’s ability to cope with the aftereffects of disasters is likely related to traits and characteristics of their families of origin. Families possess varying degrees of adaptive capacity to cope with disasters [15]; they have differential resources with respect to human, economic, social, and political capital that they can draw upon when a disaster strikes [16]. For example, in a follow-up study of Louisiana households, it was found that family exposure (e.g., a parent’s exposure) to the DHOS via physical contact, job, or income loss negatively influenced their child’s health [17]. What is less well understood is how other family characteristics may mitigate or exacerbate the impact of an oil spill on the mental health of a child.

In the wake of a disaster, individuals may suffer the loss of loved ones, health, homes, financial stability, social support, and other resources important to daily living [18]. Impacted individuals demonstrate a wide range of psychological reactivity to the same event, and children are particularly vulnerable to the stressors experienced by their parents [19–21]. For example, a qualitative study of families affected by the DHOS revealed that children suffered poor health outcomes, and in some cases toxic stress, as a result of exposure to familial stress emerging from livelihood disruptions. The toxic stress was often caused by caregivers’ displaced distress, ambiguous loss through caregivers’ physical and/or emotional absence, and children’s recognition of their families’ dire financial situations [22]. Such results lend evidence to theoretical frameworks that involve parental stress impacting parenting behaviors and subsequently leading to negative consequences for a child’s emotional well-being.

The Conservation of Resources (COR) model of stress and adaptation provides further explanation by suggesting that people are driven to acquire, preserve, or protect valued resources that are used to attain life objectives. These resources may include objects (e.g., housing), conditions (e.g., marriage), personal characteristics (e.g., self-esteem), and energies (e.g., time, money) [23,24]. Disasters often threaten or deplete these resources, leading to stress and negative mental health outcomes. Moreover, when individuals lack the resources to effectively mitigate a net loss, the losses can continue to accumulate, resulting in a “loss spiral” with accelerated negative effects [25].

The objectives of this study are to: 1) assess the effect of maternal exposure to the Deepwater Horizon Oil Spill on children’s mental health, and 2) examine whether maternal perceived loss of resources following the DHOS mediates this relationship. We hypothesize that mothers who are exposed to an oil spill experience subsequent resource loss, which negatively impacts their children’s mental health.

## Methods

### Study design, population, data collection

The Women and Their Children’s Health (WaTCH) Study is a longitudinal study in seven southern Louisiana parishes designed to assess the health effects of the Deepwater Horizon Oil Spill. Women were selected as the target population because they represent an influential yet vulnerable and understudied group. They are often central to decision-making processes within families and households, particularly with respect to decisions regarding health, support, diet, and child rearing; and they have remained relatively understudied with respect to the DHOS. Data for the present analysis were from the first and second waves of interviews of mothers and children (Wave 1: November 17, 2012-September 28, 2014; Wave 2: June 11, 2015-May 11, 2016). Details of the study are presented elsewhere [26]. Briefly, women were randomly recruited through an address-based sampling frame. Women were eligible to participate if they were between 18 and 80 years old and lived in the study area at the time of the oil spill. They were administered a 60-minute, computer-assisted telephone interview, comprised of questions on medical, social, emotional, and behavioral domains. Those who reported having a child between the ages of 10 and 17 years of age living at home at the time of the oil spill were invited to participate in further interviews of their children. One child per family was randomly selected. At Wave 1, 2852 women completed the baseline interview. Of these, 1231 also participated in the home visit, and 628 of their children were included in a child’s health substudy. At Wave 2, 2030 women and 445 children were reinterviewed. The sample for this analysis consisted of the 445 children reinterviewed at Wave 2 along with their mothers. Study data were collected and managed using Research Electronic Data Capture (REDCap) electronic data capture tools [27]. The study was approved by the Louisiana State University Health Sciences Center Institutional Review Board. Mothers provided informed oral consent via telephone, documented by study staff in REDCap (a waiver of documentation of informed consent was granted by the IRB) and then documentation of written informed consent at the in-person interview. Additional parental informed consent and child assent were obtained at the time of the in-person interview for the children.

### Measures

#### Outcomes.

Children’s mental health and psychological adjustment were assessed at Wave 2 through the Strengths and Difficulties Questionnaire (SDQ) [28], a 25-item instrument that was administered to the child by an interviewer. The SDQ asks about both positive and negative attributes, using a 3-point Likert scale to indicate to what extent each attribute applies to the target child. The 25 items are divided between five scales of 5 items each, generating scores for emotional symptoms, conduct problems, hyperactivity-inattention, peer problems, and prosocial behavior. A total difficulties score was calculated by summing the scores on the emotional symptoms, conduct problems, hyperactivity-inattention, and peer problems subscales. Higher scores signify a greater level of overall behavioral and emotional challenges.

#### Exposure.

Maternal exposure to the oil spill was measured at Wave 1 with eight dichotomous self-reported items. Two latent variables identified through Confirmatory Factor Analysis (CFA) in previous studies were identified as physical exposure to the oil spill (5 items) and economic exposure to the oil spill (3 items) [9,29,30]. Examples of physical exposure included “Oil spill caused damage to areas fished commercially” and “Oil spill directly affected recreational activities of household.” (An additional item related to property damage attributed to the spill was removed from the physical exposure latent variable due to non-significance.) An example of economic exposure included “Lost household income due to employment disruption because of oil spill.” Higher scores on each scale indicate greater oil spill exposure.

#### Mediator.

Conservation of Resources theory posits that individuals possess both internal (e.g., sense of control, self-efficacy) and external (e.g., money, time, skills) resources [31]. The possession of a broad range of resources provides a variety of coping options and a sense of psychological well-being. Disasters such as oil spills may deprive individuals of these resources, reducing coping options and resulting in psychological distress [23]. For this study, mothers’ loss of resources was measured using 23 items adapted from the Conservation of Resources-Evaluation (COR-E) instrument [24]. Participants were asked to what extent in the past six months they had lost a variety of resources. Responses ranged from “not at all (0)” to “to a great degree (4).” Exploratory Factor Analysis was conducted on the 23 measurement items, and a variety of models were tested. A 2-factor higher order model had the best fit (RMSEA = 0.057, prob RMSEA<=0.05 = 0.068) and was selected to create the mother’s resource loss latent variable, which was hypothesized and subsequently confirmed as mothers’ financial resource loss and mothers’ social resource loss subscales. A minimum factor loading of 0.4 was enforced, leaving two of the measurements not loading strongly on either factor, so they were removed. The final constructs thus used 21 of the items. Mothers’ financial resource loss subscale included 11 items such as “money for extras” and “adequate food,” while mothers’ social resource loss subscale included 10 items such as “closeness with one or more family members,” and “companionship.” Scores for each of the subscales were calculated by averaging the items for each subscale. The full list of items can be found in Table 2.

### Statistical analysis

Descriptive statistics (frequencies, proportions, means, standard deviations) for the sample population were calculated using SAS/STAT software, Version 9.4 of the SAS System for Windows. Structural equation modeling (SEM) analyses were conducted in Mplus (v7.2) [32] to test whether maternal resource loss mediates the effect of maternal oil spill exposure on children’s mental health. SEM models were estimated using the weighted least squares mean and variance adjusted (WLSMV) estimator. Oil spill exposure indicators (binary) and maternal resource loss items (ordinal) were specified as categorical variables. Under WLSMV, categorical indicators are modeled using a probit link and threshold parameterization within an underlying latent response variable framework. Estimation is based on polychoric and tetrachoric correlations, and standard errors and model chi-square statistics are mean- and variance-adjusted to provide robustness to non-normality. Children’s mental health (SDQ Total Difficulties Score) was modeled as a continuous observed outcome, and latent constructs were specified as continuous. Model fit was assessed through examination of the comparative fit index (CFI), the Tucker-Lewis Index (TLI), and the root mean square error of approximation (RMSEA). A CFI/TLI of 0.95 or greater and a RMSEA of 0.05 or lower were considered guidelines of good model fit [33]. We assessed mediation by testing for direct and indirect effects between maternal oil spill exposure and children’s mental health. Missing data were excluded. One of the assumptions for mediation analysis includes correct temporal ordering, such that the exposure occurs before the mediator and the mediator occurs before the outcome. Because the mediator and outcome are both measured at Wave 2, strict causal conclusions cannot be made. Other assumptions include no unmeasured confounders of the exposure-mediator, mediator-outcome, and exposure-outcome relationships and that these relationships are linear. The final sample size was 445 mother-child pairs.

## Results

### Participant characteristics

Table 1 shows the characteristics of the study participants at Wave 1. Among the 445 mother and child pairs included in the analysis, the majority were White (50% and 49%, respectively) with a mean age of 42.3 (SD 7.7) and 13.3 (SD 2.2) years, respectively. Most women had at least a high school education (91%), were married or living with a partner (66%), and were currently employed (65%). Over half (58%) had a pre-oil spill income over $40,000 per year.

**Table 1:** Characteristics of mothers and children, women and their children’s health study, Louisiana, N = 445*.

Characteristic	N	%
***Mothers***		
**Race/Ethnicity**		
Non-Hispanic White	194	49.62
Non-Hispanic Black	170	43.48
Hispanic/more than one race/other	27	6.91
**Education**		
Less than high school	41	9.21
High school graduate	259	58.20
College or higher	145	32.58
**Pre-Oil Spill Annual Household Income**		
Less than $20,000/yr	92	21.10
$20,001 - $40,000/yr	91	20.87
$40,001 - $60,000/yr	69	15.83
Over $60,000/yr	184	42.20
**Marital Status**		
Married/living with partner	293	65.84
Widowed/divorced/separated/never married	152	34.16
**Current Employment Status**		
Employed	290	65.32
Unemployed	154	34.68
**Age at Wave 1, years (mean, SD)**	42.29 (7.7)	27-78
***Children***		
**Race/Ethnicity**		
Non-Hispanic White	191	48.48
Non-Hispanic Black	170	43.15
Hispanic/more than one race/other	33	8.38
**Age at Wave 1, years (mean, SD)**	13.29 (2.21)	10-17

* Missing values: Mother’s race (54), Income (9), Employment (1), Mother’s Age (0), Child’s race (51), Child’s Age (2), Total Difficulties Score (4), Externalizing Score (2), Internalizing Score (3).

Table 2 presents descriptive statistics and factor loadings for each latent variable in the structural model. Mean Maternal Oil Spill Exposure was 1.83 (SD 1.61), comprised of Physical Exposure (mean 1.08, SD 1.12) and Economic Exposure (mean 0.74, SD 0.87). The mean overall Maternal Resource Loss score was 0.72 (SD 0.71), which ranged from 0 to 3.28. The mean score on the Social Resource Loss subscale was 0.67 (SD 0.76), with the highest loss occurring for closeness with one or more family members (mean 1.00, SD 1.43). The mean score on the Financial Resource Loss subscale was 0.722 (SD 0.82), with the highest losses occurring for savings or emergency money (mean 1.69, SD 1.62) and for money for extras (mean 1.60, SD 1.56). The mean for Children’s Mental Health, as measured by the Total Difficulties Scale, was 11.06 (SD 6.10), ranging from 0 to 35.

**Table 2:** Descriptive statistics and factor loadings for measurement models, women and their children’s health study, Louisiana, N = 445.

Construct and Indicators		Standardized Factor Loadings	# on figure	Missing Data
	**N (%)** ^**a**^			
**Maternal Oil Spill Exposure, W1**	1.83 (1.61)	na		0
*Physical exposure to DHOS, mean (SD)*	1.08 (1.12)	0.724		0
Worked on any oil spill cleanup	7 (1.57)	0.862	1	0
Came into physical contact with oil spill in other way	106 (24.15)	0.571	2	6
Oil spill damaged areas fished commercially	36 (8.09)	0.641	3	0
Oil spill directly affected recreational activities of household	159 (35.97)	0.633	4	3
Any smell exposure	165 (38.64)	0.607	5	18
*Economic exposure to DHOS, mean (SD)*	0.74 (0.87)	0.846		0
Lost HH income due to employment disruption	129 (29.19)	0.738	6	3
Hit harder by spill compared to others	26 (5.94)	0.771	7	7
Oil spill had a somewhat or very negative influence on household finances	176 (39.91)	0.679	8	4
	**Mean (SD)**			
**Maternal Resource Loss Caused by DHOS**	0.72 (0.71)	na		2
*Social Resource Loss*	0.67 (0.76)	0.830		0
Closeness with one or more family members	1.00 (1.43)	0.729	9	0
Good relationship with your children	0.37 (0.92)	0.712	10	0
Time with loved ones	0.94 (1.29)	0.764	11	0
Children’s health	0.39 (0.91)	0.546	12	0
Closeness or intimacy with spouse or partner	0.64 (1.23)	0.737	13	0
Closeness or intimacy with at least one friend	0.85 (1.30)	0.713	14	0
Spouse/partner’s health	0.41 (0.98)	0.606	15	0
Companionship	0.61 (1.19)	0.859	16	0
Affection from others	0.48 (1.02)	0.884	17	0
Health of family/close friends	0.98 (1.36)	0.627	18	0
*Financial Resource Loss*	0.77 (0.82)	0.888		2
Adequate Clothing	0.47 (1.06)	0.653	19	0
Adequate food	0.56 (1.16)	0.818	20	1
Stable employment	0.55 (1.19)	0.594	21	1
Providing children’s essentials	0.54 (1.07)	0.894	22	0
Extras for children	0.97 (1.37)	0.891	23	0
Money for extras	1.60 (1.56)	0.876	24	0
Understanding from your employer/boss	0.45 (1.05)	0.540	25	0
Savings or emergency money	1.69 (1.62)	0.826	26	0
Money for transportation	0.84 (1.37)	0.839	27	0
Medical insurance	0.61 (1.29)	0.566	28	0
Help with childcare	0.20 (0.80)	0.599	29	0
	**Mean (SD)**			
**Child Mental Health (Total Difficulties Scale), W2**	11.06 (6.10)	na		4
Emotional Symptoms	3.17 (2.43)	0.665		1
Conduct Problems	1.80 (1.66)	0.695		1
Hyperactivity-Inattention	3.73 (2.40)	0.578		1
Peer Problems	2.37 (1.80)	0.496		2
^a^Number and proportion of those who said “yes”				

### Structural model

**Fig 1:**
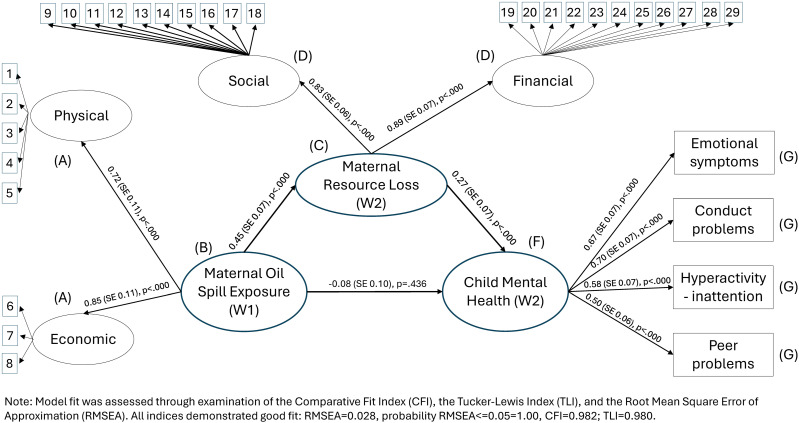
SEM depicting relationships between maternal spill exposure, maternal resource loss, and child mental health. **(A)** Physical and Economic variables identified through CFA making up the **(B)** Maternal Oil Spill Exposure Latent Variable. **(C)** Maternal Resource Loss Variable mediator variable made up of **(D)** Social and Financial Stresses as defined by EFA. **(F)** Child Mental Health latent variable calculated through the **(G)** Strengths and Difficulties Questionnaire subsections.

The structural model predicting Children’s Mental Health is shown in Fig 1. It had strong fit statistics (RMSEA = 0.028 (90% CI 0.022–0.033), CFI = 0.982, TLI = 0.980). The estimated effect of Maternal Oil Spill Exposure at Wave 1 on Children’s Mental Health at Wave 2 was −0.08 (SE 0.10), but the association was not statistically significant (p = .436). However, there was an indirect effect of maternal oil exposure on Children’s Mental Health that was consistent with mediation by Maternal Resource Loss (Maternal Oil Spill Exposure to Maternal Resource Loss: est 0.45 (SE 0.07), p < .0001; Maternal Resource Loss to Children’s Mental Health: est 0.27 (SE 0.07), p < .0001). The indirect effect of Maternal Oil Spill Exposure on Children’s Mental Health is 0.12 (SE 0.04), p < .005. That is, mothers’ increased exposure to the oil spill was associated with greater resource loss, which was associated with worse scores on children’s mental health.

## Discussion

The objectives of this study were to 1) assess the effect of maternal exposure to the Deepwater Horizon Oil Spill on children’s mental health, and 2) examine whether maternal perceived loss of resources following the DHOS mediated this relationship. We hypothesized that children’s mental health is not directly affected by their mothers’ exposure to the Deepwater Horizon Oil Spill, but that it is indirectly negatively affected when this exposure leads to maternal loss of financial or social resources. We observed a strong direct association between maternal exposure to the DHOS and maternal loss of resources, as well as between maternal resource loss and children’s mental health scores. The main direct effect of maternal exposure to the DHOS at Wave 1 did not have a significant direct effect on the mental health of the child. This suggests that maternal resource loss suggests a potential pathway through which mothers’ exposure to a disaster negatively impacts children’s mental well-being.

The mental health of both adults and children is known to be negatively impacted by disasters and by oil spills more specifically [2,4,8–11,13,14,18,29,34–37]. There is also ample evidence of the impact of parental well-being on the mental health of children [18,38–42]. The findings from the present study are consistent with this literature in suggesting a relationship between maternal oil spill exposure and worse children’s mental health. They expand the literature by identifying a route through which maternal oil spill exposure impacts children’s mental health, specifically through the loss of resources experienced by their mothers.

Hobfoll’s Conservation of Resources model provides a theoretical framework for understanding the effects of disasters on individual well-being and suggests that any event which results in actual or perceived loss of resources will produce psychological stress [23]. The types of resources considered is broad, ranging from possessions and economic resources to personal characteristics and disruption of social connections [24]. A number of studies have demonstrated associations between such resource loss and psychological impacts of disasters, including oil spills [3,31,43–48]. For example, after the Exxon Valdez Oil Spill, commercial fishers exhibited significant symptoms of depression, anxiety, and PTSD, and resource loss was related to persistence of these chronic psychological problems [3]. In particular, deterioration in relationships with others and in physical health (conditions resource loss), and income loss spirals and investments without gain (energies resource loss), were related to increased psychological symptoms. The present study is consistent with the literature in showing a relationship between disaster exposure and resource loss, specifically among mothers. It adds to and expands the literature by demonstrating that these impacts can negatively affect their children.

This study highlights the negative mental health impact of maternal financial and social resource loss on children. The role of financial resource loss on children has been well studied, particularly in the context of environmental disruptions such as floods and hurricanes [49]. That such disasters link economic resources to poor outcomes via access to social resources, among others, is less well understood. For example, a job loss may cause a family to move to a different geographic area where it has less access to social capital, a network of social relationships that help people achieve their goals [50]. This loss of social capital and decreased social involvement, whether in the form of parent-child relationships or relationships in the wider community, can result in poorer mental health outcomes for children [51,52].

Results of this study underscore the need for interventions that address both financial and social needs of child survivors of disasters such as oil spills. Calls for policy interventions that, for example, increase access to loans and other financial support to help with disaster recovery, particularly for those of low socioeconomic status, begin to acknowledge the financial needs provoked by disasters like oil spills, yet still fall short [53]. Interventions that address the social needs of survivors are also in short supply. A review of studies focusing on social capital interventions and health following disasters, for example, identified only 17 studies that met their inclusion criteria, with few that examined multilevel interventions or differential effects by subgroup [54].

Limitations of this study include the potential omission of important variables explaining the association between maternal oil spill exposure and children’s mental health, or between maternal oil spill exposure and maternal resource loss. Second, selection bias is possible in that women experiencing higher stress, lower socioeconomic status, unstable housing, or distrust of institutions may have been less likely to participate in the study. Related to this, attrition at Wave 2 of the study may have impacted study results, if participants with certain characteristics related to the exposure or outcome were less likely to have been recontacted. Third, a lack of pre-oil spill baseline data, particularly on pre-existing mental health conditions, may have influenced the strength and direction of observed associations. Fourth, all exposure and outcome data were self-reported, raising the issue of potential recall or self-report biases. Fifth, while residential mobility from one parish to another between waves was small (under 4%), there is the possibility that relocation may have resulted in misclassification or selection effects, particularly if the relocation was related to both exposure and outcome. Nevertheless, maternal oil spill exposure reflects both direct and economic impacts, rather than purely geographic proximity. As such, exposure is not contingent on continued residence near affected coastal areas at the time of the child mental health assessment. Thus, the modeled pathway is less dependent on static geographic residence at Wave 2 than on sustained material and social consequences of the spill, suggesting that any impacts would have been negligible. The maternal resource loss construct also reflects ongoing economic and psychosocial consequences that may persist regardless of residential location; relocation may, in fact, represent an additional form of resource loss that is already accounted for in that variable. Sixth, because maternal resource loss in the past six months and current child mental health were assessed at the same wave, some bidirectionality cannot be entirely ruled out, nor can mediation be definitively causally concluded. While the specific wording of these measures implies appropriate temporal sequencing of mediated events, any conclusions about mediation must be tempered. Another limitation was the inability to see if the observed relationships hold for important demographic subgroups, such as racial/ethnic or low-income populations. Finally, while the resource loss variables were reported within the past six months by the mothers, it is not possible to determine that the resource losses were caused by the DHOS; they may have been caused by other experiences. However, the significant association between maternal oil spill exposure at Wave 1 and maternal resource loss at Wave 2 lends support for a causal relationship. The study did have a number of strengths, however, including a robust sample size, the availability of longitudinal data that permitted the observation of resource loss and child mental health subsequent to maternal disaster exposure, as well as detailed information about maternal resource loss. In addition, children’s mental health was directly reported by the children themselves.

## Conclusion

This study examined the association between maternal oil spill exposure and children’s mental health among 445 mother-child pairs in southeast Louisiana following the Deepwater Horizon Oil Spill. Our findings suggest that maternal exposure to the oil spill indirectly affects children’s mental health through a pathway of financial and social resource loss that is consistent with mediation. Identifying the specific resource losses that are related to children’s mental health can point the way to targeted post-disaster interventions or pre-disaster preparedness that may mitigate poor mental health consequences.
